# A Comprehensive Update on the Management of Preterm Labor: A Narrative Review

**DOI:** 10.7759/cureus.90620

**Published:** 2025-08-20

**Authors:** Aruna Kumari Yerra, Avir Sarkar

**Affiliations:** 1 Obstetrics and Gynaecology, ESIC Medical College and Hospital, Faridabad, IND; 2 Obstetrics and Gynaecology, Noida International Institute of Medical Sciences, Noida, IND

**Keywords:** neonatal prematurity, preterm labor, preterm prelabor rupture of membranes, steroid cover in preterm labor, tocolytics

## Abstract

Preterm labor (PTL) is defined as any birth before 37 completed weeks of gestation, or fewer than 259 days since the first day of the woman's last menstrual period (LMP). It is further classified into extremely preterm (less than 28 weeks), very preterm (28 to less than 32 weeks), and moderate or late preterm (32 to less than 37 completed weeks of gestation) based on the gestational age.PTL is associated with significant neonatal morbidity and mortality and maternal morbidity. Hence, it is essential to understand the risk factors associated with it and intervene in a timely manner to prevent or reduce the adverse perinatal outcomes.

Obstetric factors, unmodifiable gynecological factors, chronic medical conditions, and recurrent periodontal disease predispose to PTL. Spontaneous PTL is mostly attributed to ascending genital tract infections during pregnancy. Maternal stress during pregnancy alters the neuroendocrine system and exaggerates the inflammatory responses and vascular hemodynamics predisposing to PTL. This narrative review has tried to summarize the various existing guidelines regarding the management (both prophylactic and therapeutic) of PTL.

## Introduction and background

Preterm labor (PTL) is defined as any birth before 37 completed weeks of gestation, or fewer than 259 days since the first day of the woman's last menstrual period (LMP). It is further classified into extremely preterm (less than 28 weeks), very preterm (28 to less than 32 weeks), and moderate or late preterm (32 to less than 37 completed weeks of gestation) based on the gestational age [1]. PTL is associated with significant neonatal morbidity (deleterious effect on the physical, mental, and psychological development of the newborn) and mortality, and maternal morbidity. Hence, it is essential to understand the risk factors associated with it and intervene timely to prevent or reduce the adverse perinatal outcomes.

Obstetric factors (multifetal gestation, preeclampsia, urinary tract infections), unmodifiable gynecological factors (prior history of PTBs, short cervical length), chronic medical conditions (gestational hypertension, diabetes mellitus), chronic smoking [2], and recurrent periodontal diseases predispose to PTL [3]. Approximately one-fourth of spontaneous PTL (sPTL) are due to ascending genital tract infections during pregnancy [4]. Maternal stress during pregnancy alters the neuroendocrine system and exaggerates the inflammatory responses and vascular hemodynamics predisposing to sPTL [5]. 

Several theories have proved that ascending genital tract infections by decidual stimulation, synthesis, and release of proinflammatory cytokines (IL-1b, IL-6, TNF-alpha, granulocyte colony-stimulating factor, or tumor necrosis factor-a) and chemokines (IL-8, MCP-1), increased the prostaglandin production, stimulate the synthesis and activity of COX-2, trigger uterine myometrial contractions, and ultimately lead sPTL [6]. Inflammatory conditions like periodontitis, pyelonephritis, pancreatitis, or genital tract conditions (bacterial vaginosis, chorioamnionitis or intra-amniotic infections) during pregnancy, due to an exaggerated immune response and increased inflammatory cytokines, elastases and MMPs induce the functional withdrawal of progesterone, a hormone that plays a significant role in maintaining pregnancy and thus leading to PTL [7]. The current comprehensive review brings to light the updated management for the prevention and treatment of PTL.

## Review

The various strategies for the prevention of PTL are as follows:

Role of progesterone

Many interventions have come up in managing a singleton pregnancy with a short cervix and no prior history of PTL (intramuscular/vaginal progesterone, cervical cerclage). Studies on preventing PTL using progesterone have shown conflicting results. A large multicenter trial demonstrated that weekly intramuscular injections of 17-OH progesterone caproate (17-OHP) in women with a short cervix between 16 and 22 weeks reduced the risk of PTL without affecting the neonatal outcomes [8]. Vaginal micronized progesterone (200 mcg daily) when used in an asymptomatic woman with a cervical length of ≤20 mm before 24 weeks of gestation and with no prior history of PTL was associated with a 44% reduction in spontaneous preterm delivery when compared to the placebo group. (RR = 0.56; 95% CI, 0.36 to 0.86) [9]. A meta-analysis of 974 singleton pregnancies with a second-trimester cervical length ≤25 mm treated with vaginal progesterone, demonstrated not only a reduction in preterm birth at <28, < 32, <34, and <37 weeks of gestation but also a 41% reduction in neonatal morbidity and mortality (RR 0.59, 95% CI 0.38-0.91) [10]. Currently, the American College of Obstetricians and Gynecologists (ACOG) recommends vaginal progesterone for preventing PTL in pregnant women with a short cervix [3]. The Society for Maternal-Fetal Medicine (SMFM) recommends intramuscular administration of 250 mg 17-OHP weekly, from 16 to 20 weeks of gestation until 36 weeks of gestation or delivery for a woman with a singleton gestation and a history of prior PTB. Vaginal progesterone is considered a reasonable alternative when 17-OHP is not available [11]. Vaginal progesterone is not recommended in multifetal gestation as it does not reduce the incidence of PTL [12]. 

Role of cervical cerclage

For ages, cervical cerclage has been done to correct the structural defects or weakening associated with a short cervix in high-risk women. In women with a prior history of PTL and a cervical length of ≤25mm, cerclage decreases preterm deliveries and perinatal deaths [13]. Studies comparing the results of cerclage over progesterone therapy and the role of combined therapy (cerclage and progesterone) in preventing PTL are lacking. The role of cervical cerclage in women with a short cervix with no prior history of preterm delivery is debatable. Prophylactic cerclage in multifetal pregnancies is not recommended as it is found to be associated with a two-fold increased risk of preterm delivery (RR = 2.2; 95% CI, 1.2 to 4) [14].

Diet in preventing preterm labor

Since poor maternal nutrition preconceptionally and during early pregnancy is found to be associated with PTL, specific nutrient intervention strategies may help in reducing the risk of PTL [15]. It is found that a “prudent” dietary pattern (consumption of good amounts of vegetables, salad, fruit and nuts, vegetable oils, water, whole grain cereals, and fiber-rich bread, and low intake of processed meat products, white bread) was associated with significant reductions in PTL risk (by 12%). The theory behind it is that a low-fat diet (due to increased antioxidant content) has an anti-stressor effect on the hypothalamic-pituitary-adrenal axis and has an anti-inflammatory effect [16]. There is sufficient evidence to show that omega-3 fatty acid supplementation during pregnancy through their anti-inflammatory and relaxant effect on uterine myometrium (increased production of prostacyclin PGI2 and PGI3) reduces the risk of early PTL [17]. Zinc supplementation by reducing maternal infections, vitamin D by its anti-inflammatory and immunomodulatory action, and magnesium sulphate through its tocolytic effects, are found to reduce the risk of PTL [18-20]. There is insufficient data to prove the efficacy of oral probiotics for preventing PTL [21]. 

Other strategies

A prospective, randomized, double-blinded multicenter study (ASPIRIN trial) in six developing countries showed that daily administration of 81 mg aspirin from/before 14 weeks of gestation lowered the preterm birth rate in nulliparous women without prior medical conditions [22]. 

After reviewing the various preventive strategies, we searched the literature to assess the various treatment modalities that are prevalent in PTL: 

Role of antibiotics in preterm labor

Previous literature showed that antibiotic administration resulted in significant prolongation of pregnancy in women with pre-labor rupture of membranes (PROM) [23]. A prospective randomized controlled double-blind study in 1986 found that the duration of pregnancy was prolonged by 32.5 days (in women with preterm uterine contractions before 34 weeks of gestation) receiving antibiotics (333 mg of erythromycin orally thrice daily for seven days) when compared to 22.4 days in those not receiving them [24]. Other studies like the randomized placebo-controlled trial of Florida (n = 150) [25], South African multicentric study (n = 43) [26], Baltic-Scandinavian randomized controlled trial (n = 59) [27], and a Danish multicentric trial (n = 112) conducted on women with PTL [28], all showed that antibiotic administration prolonged the duration of pregnancy, reduced the infant morbidity (hyaline membrane disease, pneumonia, septicemia, lower NICU admission rates) and decreased the maternal morbidity (histological chorioamnionitis and puerperal endometritis-myometritis). However, recent studies (ORACLE II) conducted on women with preterm uterine contractions showed that antibiotic administration did not significantly prolong the pregnancy and neonatal outcomes but lowered the maternal infection rates [29]. Currently, the World Health Organization (WHO) and the National Institute for Health and Care Excellence (NICE) do not recommend routine antibiotic administration in PTL with intact amniotic membrane and no clinical evidence of infection [30,31]. However, prophylactic antibiotic administration is recommended in expectants with preterm PROM and expectants with group B streptococci carrier status. The Cochrane Systematic Review of 2011 also supports these findings [32]. 

Tocolytics and preterm labor

The primary objective of tocolytic therapy is to prolong pregnancy for at least 48 hours (acute tocolysis) from the onset of PTL, to enable the administration of antenatal corticosteroids for fetal lung maturity, magnesium sulfate for fetal neuroprotection, and to transfer the mother to a tertiary care facility. Tocolysis given beyond 48 hours of PTL is called maintenance tocolysis [32]. Patient characteristics (gestational age, if neuroprotection is required), drug metabolism and their safety in pregnancy, and comorbid conditions (magnesium sulfate contraindicated in women with deranged kidney functions), must be considered while selecting tocolysis in PTL.

Betamimetics, calcium channel blockers, magnesium sulfate, and prostaglandin inhibitors are the commonly used tocolytic agents. Betamimetics stimulate the β2 receptors, increase the cyclic AMP, deplete intracellular calcium levels, and thus diminish myometrial contractility. Systematic review and meta-analysis of tocolytic therapy from 95 randomized controlled trials concluded that β2-agonist administration prolonged the pregnancy by at least 48 hours with no improvement in neonatal outcomes. Terbutaline 0.25 mg given subcutaneously and repeated every four hours is the common betamimetic for tocolysis. Maternal tachycardia, hypotension, bronchodilatation, palpitations, shortness of breath, tremors, headache, and nasal congestion are a few side effects anticipated while using terbutaline. Pulmonary edema and symptomatic arrhythmias are serious side effects demonstrated to date. The drug is contraindicated in women with heart disease, hemorrhage, or hypovolemia. If maternal infusion is not discontinued two hours or more before delivery, neonatal hypoglycemia, hypocalcemia, and ileus may occur [33]. Once the PTL stops maintenance, tocolysis with a terbutaline pump does not appear to prolong the pregnancy or improve the neonatal outcomes [34]. 

Calcium channel blockers (nifedipine, nicardipine), by preventing the calcium influx into the myometrial cells, cause inactivation of myosin light chain kinase and inhibit myometrial contractions. Routinely, nifedipine is administered orally or sublingually in a loading dose of 10-30 mg, repeated every 15-20 minutes in the first hour, followed by 10-20 mg orally every four to eight hours for tocolysis [35]. Flushing and hypotension are common side effects of sublingual nifedipine [36]. A Cochrane review of 12 randomized controlled trials (n = 1029 women) showed that nifedipine, compared to ritodrine, is more effective in prolonging pregnancy beyond seven days with fewer maternal side effects [37], while another study revealed no such differences in tocolytic efficacy [38]. 

Magnesium sulfate inhibits myometrial contractions by competing with calcium at plasma membrane voltage-gated channels, prevents the influx of intracellular calcium, and prevents activation of myosin light chain kinase. A loading dose of 4-6 g in 10-20% distilled water given over 30 minutes, followed by a continuous infusion of 2 g/hour, is the common regimen [39]. The infusion rate is titrated to a maximum of 4 g/hour or until <1 contraction/10 minutes are noted. Respiratory depression and cardiac arrhythmias are the serious side effects noted. Calcium gluconate is the antidote for magnesium sulfate toxicity. Its use has been popularized due to the additional neuroprotective effect. In 1997, a double-blinded, placebo-controlled randomized clinical trial (BEAM trial) was conducted, including pregnant women between 24 and 31 weeks and six days of gestation with ruptured membranes, and women in advanced PTL. The women satisfying the inclusion criteria were allocated to the magnesium sulfate or the placebo group. Magnesium sulfate was given 6 g intravenously as a loading dose, followed by 2 g/hour intravenously for up to 12 hours. The same was discontinued when delivery was not imminent after 12 hours and restarted if it recurred before 33 weeks, six days. It was found that the rate of moderate to severe cerebral palsy was significantly lower in the magnesium group (1.9% vs. 3.5%) in the placebo group (RR = 0.55, 95% CI: 0.32-0.95) [40]. The joint consensus of ACOG (upper gestational age limit-31 weeks and six days) and the SMFM recommends antenatal administration of magnesium sulphate for a threatened or imminent PTL at or beyond 24 weeks of gestation [41]. Haas et al. in 2012 conducted a meta‐analysis of 55 randomized controlled trials and found magnesium sulfate to be effective in delaying birth by 48 hours (short-term tocolysis) [42]. 

Indomethacin is a prostaglandin inhibitor (suppresses the myometrial gap junctions, decreases the free intracellular calcium levels, inactivates myosin light chain kinase, and thus reduces the myometrial contractility). The network meta-analysis conducted by Haas et al. in 2012 found that prostaglandin inhibitors, when compared to a placebo, are more effective in delaying delivery by 48 hours (odds ratio 5.94, 95% credible interval 2.14-12.34) [42]. Loading dose of 50 mg of Indomethacin followed by 25-50 mg orally every six hours for up to 48 hours acts as a tocolytic. Both human and animal models have shown that indomethacin crosses the placenta and may lead to premature closure of the ductus arteriosus, oligohydramnios, necrotizing enterocolitis, and intraventricular hemorrhage (IVH) [43]. 

Studies on animal models showed that nanoparticles LIP-IND-ORA (liposome carrying indomethacin with oxytocin receptor antagonist on the surface), when delivered to pregnant mice, reduced the uterine contractions, preterm birth, and also the placental passage of the drug to the fetus [44]. In vivo animal study by Bariani et al. demonstrated that resveratrol, a naturally occurring polyphenol, prevented lipopolysaccharide-induced PTL through its anti-inflammatory property and showed a tocolytic effect by downregulating COX-2, changing the uterine prostaglandin and endocannabinoid profiling [45]. 2-aminoethoxydiphenyl borate (2-APB), glycyl-H-1152 dihydrochloride (GH), and HC-067047 are the three novel tocolytics reported in a comprehensive ex vivo study conducted by Hossain et al in 2022 [46]. 

Antenatal corticosteroids and preterm labor

One of the greatest evolutions in the field of obstetrics is the use of antenatal corticosteroids to reduce the neonatal morbidity and mortality associated with PTL. Dexamethasone and betamethasone are the most commonly used drugs. Both drugs act on fetal lungs, increasing protein production, biosynthesis of phospholipids, and the appearance of surfactants [47]. The Cochrane Systematic Review and meta-analysis on “Antenatal corticosteroids for accelerating fetal lung maturation” (including 21 randomized trials, 3885 women and 4269 infants) concluded that both dexamethasone and betamethasone are beneficial with no difference between them in reducing the fetal respiratory distress syndrome, IVH, and long-term neurological sequelae associated with PTL [48,49]. A single course of antenatal corticosteroids is indicated in all pregnant women between 24 and 34 weeks of gestation who are at risk of preterm delivery within seven days [50]. 

Currently, the total dose of antenatal corticosteroids recommended to have a maximal benefit on fetal lung maturity is 24 mg. The regime recommended by the (International Federation of Gynecology and Obstetrics (FIGO) 2021) is two doses of 12 mg betamethasone/dexamethasone intramuscularly 24 hourly as against the four-dose regime of dexamethasone (6 mg intramuscularly 12 hourly) as recommended by NIH 1995 [51,52]. There is also a controversy over whether to follow a single course or a repeat course regime. Since the effect of antenatal corticosteroids lasts only for seven days, earlier it was recommended (Cochrane Review by Crowther 2015) to repeat the dose when the pregnancy prolongs beyond that period. It has been found that a repeat dose antenatal corticosteroid regime has short-term effects on the mother and fetus. There is an increased risk of infection, endometritis, and chorioamnionitis in mothers with preterm PROM and a risk of low birth weight and early-onset neonatal sepsis in fetuses [53]. A study by Deren et al. [54] showed that maternal betamethasone administration led to a transient but significant reduction in biophysical profile scores without changes in middle cerebral and umbilical artery Doppler indices. Hence, the latter may be used for surveillance of fetuses previously exposed to the antenatal steroids [54]. A longitudinal study by Weiss et al. [55] demonstrated that infants whose mothers received antenatal corticosteroids had significantly lower resting state and post-stressor cortisol levels across the first year of life (due to persistent hypo-arousal of their hypothalamic-pituitary-adrenal (HPA) axis) than infants whose mothers did not receive antenatal corticosteroids. Synthetic glucocorticoids (betamethasone and dexamethasone) fail to get converted to inactive metabolites (cortisone) by 11-βHSD2, cross the placental barrier, and bring alterations in fetal HPA axis, thus suppressing hormone production (cortisol) [55]. 

Role of rescue cerclage in preterm labor

Cervical insufficiency (inability of the cervical tissue to hold back the products of conception) can lead to prolapse of the amniotic membrane outside the cervix and vagina and accounts for 5-15% of second-trimester pregnancy losses [56]. Rescue cervical cerclage (reducing the amniotic membrane and tightening the expanded cervix) is performed to avoid pregnancy losses. The procedure is invasive and can result in premature rupture of membranes or ascending infection. Subclinical microbial invasion of the amniotic cavity or intra-amniotic inflammation are contraindications for the procedure [57]. A systematic review (96 studies, and 3239 women), comparing the effectiveness of rescue cervical cerclage with expectant management in the prevention of extremely premature birth, showed that the former procedure led to a significant prolongation of pregnancy and reduction in delivery at <28 weeks of gestation in both singleton and twin pregnancies [58]. The degree of cervical dilatation at diagnosis, the total leucocyte count, CRP levels in the maternal peripheral blood, and IL-6 levels in the amniotic fluid are the factors that contribute to the success of rescue cerclage [59]. Currently, the ACOG (for cervical insufficiency in the second trimester of pregnancy), the Royal College of Obstetricians and Gynecologists, and the Society of Obstetricians and Gynecologists of Canada (for dilatations of <4 cm irrespective of gestation) recommend rescue cerclage for threatened PTL [60,61]. 

## Conclusions

Although it is not possible to prevent PTL, careful measures can be adopted to lower the risks associated with it. This comprehensive narrative review has tried to address both the preventive and therapeutic aspects of PTL. Further statistical correlations in the form of meta-analysis can be instrumental in justifying the rationale behind these therapeutic strategies. 
